# MoS_2_ Nanosheets Decorated with Fe_3_O_4_ Nanoparticles for Highly Efficient Solar Steam Generation and Water Treatment

**DOI:** 10.3390/molecules28041719

**Published:** 2023-02-10

**Authors:** Zhi Bai, Haifeng Xu, Guang Li, Bo Yang, Jixin Yao, Kai Guo, Nan Wang

**Affiliations:** 1 School of Mechanical and Electronic Engineering, Suzhou University, Suzhou 234000, China; 2 School of Information Engineering, Suzhou University, Suzhou 234000, China; 3 Anhui Key Laboratory of Information Materials and Devices, Institute of Physical Science and Information Technology, School of Materials Science and Engineering, Anhui University, Hefei 230601, China; 4 Key Laboratory of Structure and Functional Regulation of Hybrid Materials of Ministry of Education, Institute of Physical Science and Information Technology, School of Materials Science and Engineering, Anhui University, Hefei 230601, China; 5 School of Physics and Electronic Information, Huaibei Normal University, Huaibei 235000, China; 6 Universities Joint Key Laboratory of Photoelectric Detection Science and Technology in Anhui Province, Hefei Normal University, Hefei 230601, China; 7 Anhui Provincial Engineering Laboratory on Information Fusion and Control of Intelligent Robot, Wuhu 241002, China

**Keywords:** solar steam generation, Fe_3_O_4_, MoS_2_, desalination, water treatment

## Abstract

The shortage of water resources has always been one of the most difficult problems that perplexes humanity. Solar steam generation (SSG) has been a new non-polluting and low-cost water purification method in recent years. However, the high cost of traditional photothermal conversion materials and the low efficiency of photothermal conversion has restricted the large-scale application of SSG technology. In this work, composite materials with Fe_3_O_4_ nanospheres attached to MoS_2_ nanosheets were synthesized, which increased the absorbance and specific surface area of the composite materials, reduced the sunlight reflection, and increased the photothermal conversion efficiency. During the experiment, the composite material was evenly coated on cotton. The strong water absorption of cotton ensured that the water could be transported sufficiently to the surface for evaporation. Under one sun irradiation intensity, the evaporation rate of the sample synthesized in this work reached 1.42 kg m^−2^ h^−1^; the evaporation efficiency is 89.18%. In addition, the surface temperature of the sample can reach 41.6 °C, which has far exceeded most photothermal conversion materials. Furthermore, the use of this composite material as an SSG device for seawater desalination and sewage purification can remove more than 98% of salt ions in seawater, and the removal rate of heavy metal ions in sewage is close to 100%, with a good seawater desalination capacity and sewage purification capacity. This work provides a new idea for the application of composite materials in the field of seawater desalination and sewage purification.

## 1. Introduction

Since the twentieth century, with the continuous growth of the global population and the development of industry, although water resources on the earth are very abundant, freshwater resources only account for 2.5%. The shortage of freshwater resources has gradually become one of the problems that people need to solve urgently [1,2]. Efficient, inexpensive, and convenient access to freshwater resources is the goal pursued by numerous scholars [3,4], that can be used directly by humans, which has attracted the attention of many scholars [5].

The high-efficiency strategy of the SSG system is to improve the rate of water evaporation; the main ways to accomplish this are as follows: (1) Increase the rate of water transportation [6]. (2) Improve the photothermal conversion efficiency of photothermal conversion materials [7]. (3) Improve the light absorption performance of photothermal conversion materials. (4) Reduce the heat loss of the system and reduce the salt deposition on the evaporation surface. In response to the above key points, many scholars have proposed approaches to solve them. For example, Tao et al. reported a semiconductor composite material based on 3D CuS nanoflowers. Nanoflower composites with 3D morphology can be in contact with more sunlight, increasing the photothermal conversion efficiency of the materials [8]. Kim et al. used Ni to deposit a self-aggregating alumina nanowire structure to prepare a broadband solar energy absorption surface, and the light absorption property of the material is then improved [9]. He et al. made a renewable SSG material based on cellulose composite aerogels with cellulose nanofibers as the skeleton and polyethylene imine as the binder. The material has a strong water absorption, which can ensure sufficient water transmission to the evaporation interface [10]. Li et al. prepared a unique Janus photothermal material using Fe_3_O_4_ nanoparticles and carbon aerogel. In that aerogels can provide a fast water supply, Fe_3_O_4_ has good photothermal conversion properties, and the material also effectively retards salts deposition [11]. Many scholars have proposed methods to improve the water evaporation rate, few have achieved a high evaporation rate, low cost, and green environmental protection at the same time. Therefore, this work is focused on the synthesis of a material with high water evaporation performance and low cost.

Due to its high stability, low thermal conductivity, and high water absorption, cotton is an ideal material for water transmission and heat insulation. Under the effect of the capillary phenomenon, water will be transported through cotton to the water evaporation interface, and will soon be transported to the entire light receiving layer. Also, because cotton has good thermal insulation, it will separate sunlight from water, prevent sunlight from contacting directly with water, and improve the efficiency of light–heat conversion of materials [12]. In terms of photothermal conversion materials, MoS_2_ has quickly become the object of many scholars’ research because of its good photothermal conversion performance, mechanical properties, large specific surface area, good stability, strong adsorption, and low price. The research shows that MoS_2_ has good light absorption performance, and can absorb 5–10% of the sun’s light in a single layer of MoS_2_ with a thickness of less than 1 nm [13,14]. Although MoS_2_ has a strong absorbance, its surface morphology is similar to graphene and is layered, so it will reflect sunlight to some extent and reduce the absorption rate of sunlight. Furthermore, MoS_2_ has a high rate of photogenerated carrier recombination, and the sunlight absorbed by MoS_2_ will be partially converted into chemical energy, so the growth of nanoparticles in MoS_2_ semiconductor materials is conducive to improving its photothermal conversion efficiency [15]. For example, Zhang et al. used the hydrothermal method to achieve Zn-doped MoS_2_ to prepare Zn-MoS_2_ to achieve higher solar absorption, and then used Zn-MoS_2_ to improve the evaporation performance of sorghum straw, which has a high innovation and practical prospect [16]. Fe_3_O_4_ is a typical narrowband semiconductor with a strong photothermal conversion efficiency in the visible band. If Fe_3_O_4_ is grown on the surface of MoS_2_ to prepare Fe_3_O_4_@MoS_2_ composite material, the combination of the N-type narrowband gap semiconductor Fe_3_O_4_ and MoS_2_ will further broaden the optical conversion wavelength range of MoS_2_ and improve sunlight utilization [17,18,19]. The N-type semiconductor Fe_3_O_4_ and the P-type semiconductor MoS_2_ will form a P-N heterostructure at the contact point, which can improve the photothermal conversion performance of the composite [20,21,22,23].

Here, we report the hydrothermal synthesis of Fe_3_O_4_@MoS_2_ materials for seawater desalination and sewage treatment. The material was applied uniformly to the cotton surface as a photothermal conversion layer of the SSG for desalination and sewage purification. Using a hydrothermal method to grow Fe_3_O_4_ nanospheres on MoS_2_ nanosheets, two kinds of particles would form a P-N heterostructure at the contact point, enhancing the performance of photothermal conversion. Furthermore, using cotton as a support for the photothermal conversion material, hydrophilic cotton and hydrophobic Fe_3_O_4_@MoS_2_ composed the photothermal conversion layer, cotton hydrophilicity provided a path for water and salt diffusion, and hydrophobic Fe_3_O_4_@MoS_2_ played a role in hindering water and thermal insulation. Simulated seawater with a mass fraction of 3.5 wt% was used as the water source to be desalinated. Under one sun irradiation, the evaporation rate was 1.42 kg m^−2^ h^−1^, and the evaporation efficiency was 89.18%. This SSG device can almost completely remove heavy metal ions, soluble organics, and oils from seawater and sewage.

## 2. Results and Discussion

### 2.1. Characterization of Samples

**Figure 1:**
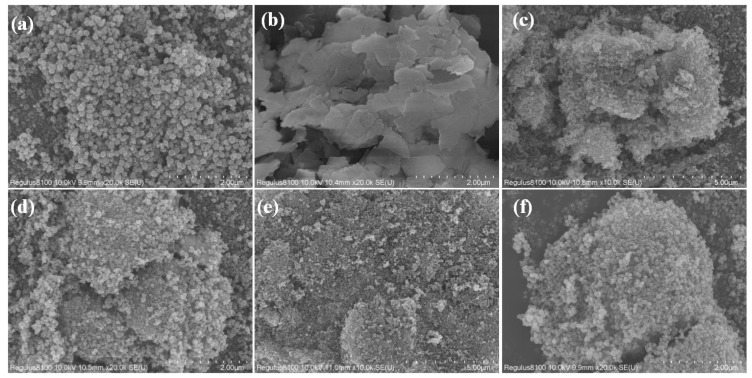
SEM of (**a**) Fe_3_O_4_ at 20k times, (**b**) MoS_2_ at 20k times, (**c**) Fe-Mo-1 at 10k times, (**d**) Fe-Mo-1 at 20k times, (**e**) Fe-Mo-2 at 10k times and (**f**) Fe-Mo-2 at 20k times.

The morphology and microstructure of Fe_3_O_4_, MoS_2_, Fe-Mo-1, and Fe-Mo-2 observed by SEM are shown in Figure 1a–f. Figure 1a shows the microscopic images of Fe_3_O_4_ magnified 20k times. It can be seen that the nano-Fe_3_O_4_ sample prepared in this work is spherical, the diameter of the spheres is not uniform, the average is about 200 nm, the surface of the nanospheres is rough, and the compact between the nanospheres makes the comparison area of the samples smaller. Figure 1b shows the microscopic image of MoS_2_ magnified 20k times. It can be seen that MoS_2_ is flaky with a smooth surface and each piece is irregular in shape and size. Figure 1c,d shows the microscopic images of the Fe-Mo-1 sample magnified 10k and 20k times, respectively. It can be found that Fe_3_O_4_ nanospheres were grown on MoS_2_ nanosheets and the density of the nanospheres decreased so that more surfaces of the nanospheres were exposed, making the comparative area of the Fe-Mo-1 sample increase and sunlight be more easily absorbed, so this sample has a higher potential for solar absorbance [24]. Figure 1e,f shows the microscopic images of sample Fe-Mo-2 magnified 10k and 20k times, respectively. The figure shows that the nanoparticles on the surface of the sample are denser compared to those of Figure 1c,d, which is mainly due to the higher Fe_3_O_4_ content in the reactants.

**Figure 2:**
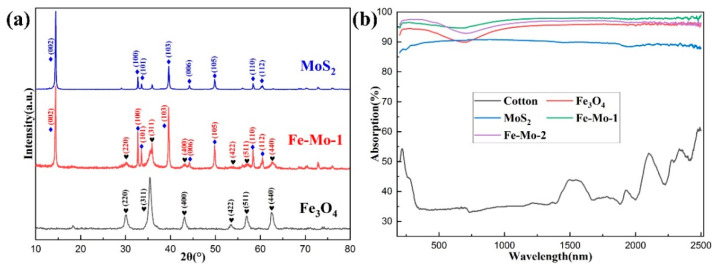
(**a**) XRD peaks of Fe_3_O_4_, MoS_2_, and Fe-Mo-1. (**b**) Optical absorption spectra of cotton, Fe_3_O_4_, MoS_2_, Fe-Mo-1, and Fe-Mo-2.

The XRD patterns of the Fe_3_O_4_, MoS_2_, and Fe-Mo-1 samples are shown in Figure 2a. The diffraction peaks of Fe_3_O_4_ prepared in this work appear mainly at 30.12°, 35.48°, 43.12°, 53.50°, 57.03°, and 62.62°, corresponding to the characteristic peak planes of (220), (311), (400), (422), (511), and (440), respectively (JCPDS: 88-0866). The diffraction peaks of MoS_2_ appear mainly at 14.40°, 32.70°, 33.52°, 39.57°, 44.18°, 49.82°, 58.36°, and 60.45°, respectively, corresponding to (002), (100), (101), (103), (006), (105), (110), and (112) characteristic peak planes (JCPDS: 87-2416). The characteristic peaks of Fe_3_O_4_ and MoS_2_ appear in the XRD patterns of the Fe-Mo-1 composite sample, which indicates that Fe_3_O_4_ and MoS_2_ are contained in the sample. As can be seen by the characteristic peak contrast, the intensity of the characteristic peak of MoS_2_ was slightly enhanced. This may be due to the absence of water inclusion in the MoS_2_ crystals after the hydrothermal reaction, which makes the crystals tighter and diffract more strongly between each other. There is no obvious change in the characteristic peaks of Fe_3_O_4_ [25]. The solar absorption rate of the samples directly determines the working efficiency of the SSG device. A UV-Vis-NIR spectrometer is used to determine the solar absorption rate of Fe-Mo-1, Fe-Mo-2, Fe_3_O_4_, MoS_2_, and cotton, and the measurement results are shown in Figure 2b. The absorption rates of Fe-Mo-1, Fe-Mo-2, Fe_3_O_4_, MoS_2_, and cotton at 200–2500 nm are approximately 97.11%, 96.19%, 94.6%, 89.71%, and 39.76%, respectively. The absorption rates of MoS_2_ and Fe_3_O_4_ were similar to those of the references [20,26]. As expected, both MoS_2_ and Fe_3_O_4_ showed high absorption. The Fe_3_O_4_ absorption rate can be found to be better than that of MoS_2_. This may be because the surface of the Fe_3_O_4_ nanosphere is rough, while the surface of the MoS_2_ nanosheet is smooth, which will reflect some of the sunlight. However, the light absorption of the Fe-Mo-1 and Fe-Mo-2 samples is better than that of Fe_3_O_4_ and MoS_2_; this is mainly due to the fact that Fe_3_O_4_ nanospheres are attached to the surface of MoS_2_ nanosheets, which increases the specific surface area of the sample and increases the contact area with sunlight, thus increasing the light absorption rate. The light absorption of Fe-Mo-1 is slightly better than that of Fe-Mo-2, which is mainly caused by the different content of Fe_3_O_4_ attached to the surface of MoS_2_. The author has done many experiments and found that it is not the higher the Fe_3_O_4_, the better the light absorption of the composite, nor the higher the MoS_2_, the better the light absorption of the composite, but the existence of the best proportion that can make the composite achieve the best light absorption.

### 2.2. Photothermal Conversion Performance Test

**Figure 3:**
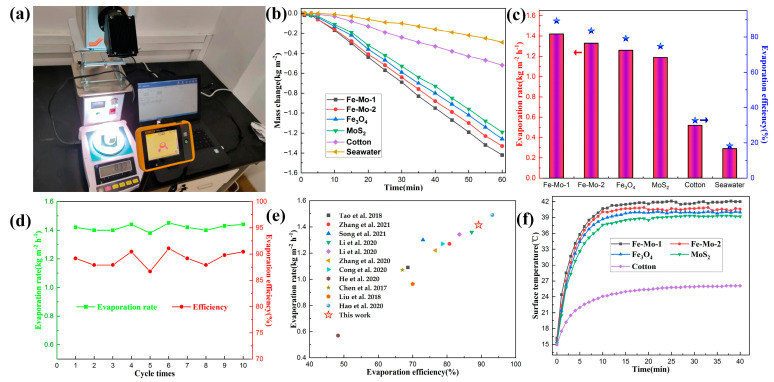
(**a**) Physical picture of the SSG test device in the laboratory. (**b**) Variation of the change in seawater mass with time for different samples under one sun irradiation. (**c**) Seawater evaporation rate and efficiency of different samples under one sun irradiation (The bar graph corresponds to the evaporation rate on the left axis and the pentagram corresponds to the evaporation efficiency on the right axis). (**d**) Cyclic experiment of the Fe-Mo-1 sample under one sun irradiation. (**e**) Comparison of the evaporation rate and efficiency of the Fe-Mo-1 with other literature (The top to bottom plots correspond to refs. [8,12,19,27,28,29,30,31,32,33,34], respectively). (**f**) Trend chart of surface temperature changes over time for different samples during the SSG test.

The physical picture of the SSG test device in the laboratory is shown in Figure 3a. The evaporation performance of Fe-Mo-1, Fe-Mo-2, Fe_3_O_4_, MoS_2_, cotton, and seawater samples was tested, and the evaporation performance of all samples was analyzed and compared. The test results are shown in Figure 3b. It can be clearly seen from the figure that, under one sun irradiation, after one hour’s test, the mass change of pure seawater and cotton is less, which is 0.29 kg m^−2^ and 0.52 kg m^−2^, respectively. When Fe_3_O_4_ and MoS_2_ samples are used for the tests, the mass change is increased to a certain extent; they are 1.26 kg m^−2^ and 1.19 kg m^−2^, respectively. The mass change of Fe-Mo-1 and Fe-Mo-2 is greater than that of pure Fe_3_O_4_ and pure MoS_2_, which are 1.42 kg m^−2^ and 1.33 kg m^−2^, respectively. According to the calculation method described in Formula (1), the evaporation rates of several samples can be obtained in descending order as follows: Fe-Mo-1 (1.42 kg m^−2^ h^−1^), Fe-Mo-2 (1.33 kg m^−2^ h^−1^), Fe_3_O_4_ (1.26 kg m^−2^ h^−1^), MoS_2_ (1.19 kg m^−2^ h^−1^), cotton (0.52 kg m^−2^ h^−1^), and seawater (0.29 kg m^−2^ h^−1^). Furthermore, according to Formula (2), the evaporation efficiency of four samples can be calculated as follows: Fe-Mo-1 (89.18%), Fe-Mo-2 (83.52%), Fe_3_O_4_ (79.13%), MoS_2_ (74.73%), cotton (32.66%) and seawater (18.21%) (Figure 3c). To verify the stability of the photothermal conversion performance of the prepared Fe-Mo-1 sample, 10 cyclic tests were performed under the condition that the external conditions remained unchanged, as shown in Figure 3d. The average evaporation rate and evaporation efficiency of the 10 experiments were respectively obtained as 1.418 kg m^−2^ h^−1^ and 89.1%. It can be seen that the photothermal conversion performance of the sample is relatively stable. On the basis of the above data, it can be seen that the Fe_3_O_4_@MoS_2_ produced in this work has better water evaporation performance than pure Fe_3_O_4_ and pure MoS_2_, and higher photothermal conversion performance under the same irradiation intensity. Figure 3e shows the comparison of the evaporation efficiency and evaporation rate of this work with different literature under one sun irradiation. It can be seen that this work is better than most reported studies [8,12,19,24,27,28,29,30,31,32,33].

**Figure 4:**
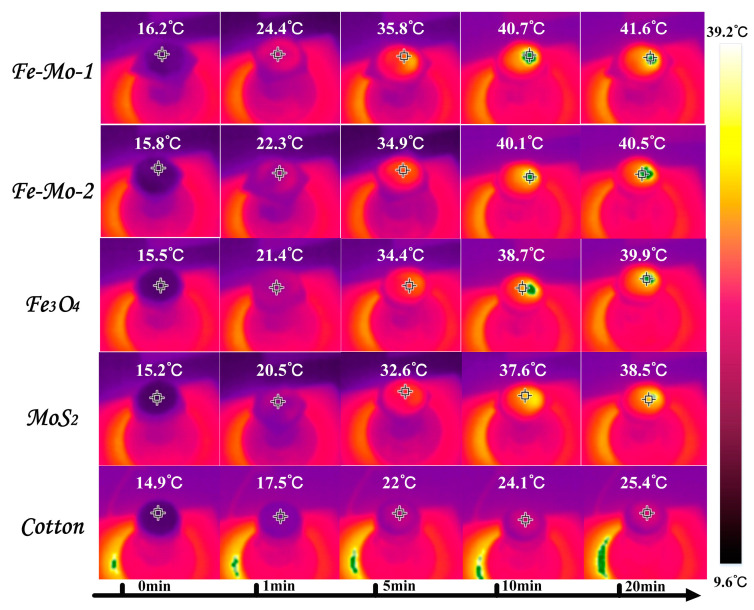
Infrared radiation images of different samples at different times in the test process.

In order to measure the trend of surface temperature variation of different samples under one sun irradiation, the photothermal conversion performance of the samples was reflected. In the evaporation experiment of different samples, the infrared radiation imager is used to record the temperature changes on the surface of the samples, and the curve of the changes is shown in Figure 3f. In the figure, the temperature of the cotton surface is the slowest and the surface temperature of the other four samples rises faster. In the first five minutes, the surface temperature of Fe-Mo-1, Fe-Mo-2, Fe_3_O_4_, MoS_2_, and cotton increased by 19.6 °C, 19.1 °C, 18.9 °C, 17.4 °C, and 7.1 °C, respectively. The first three samples have the highest temperature increase, including Fe_3_O_4_, which shows that Fe_3_O_4_ has good photothermal conversion performance. The temperature of all samples began to rise relatively slowly between 5 min and 10 min. The temperature of the first four samples increased by about 5 °C on average, while the temperature of cotton rose less, by about 2 °C. After 10 min, the surface temperature of all samples began to become stable. When the temperature of the sample surface is stable, it can be found that the surface temperature of the Fe-Mo-1 sample is higher than that of Fe-Mo-2, Fe_3_O_4_, MoS_2_, and cotton by 1.4 °C, 2 °C, 2.8 °C, and 15.9 °C, respectively [34]. Figure 4 shows the infrared imaging of five different samples under one sun radiation for 0 min, 1 min, 5 min, 10 min, and 20 min. The above data confirmed the good photothermal conversion performance of Fe-Mo-1.

**Figure 5:**
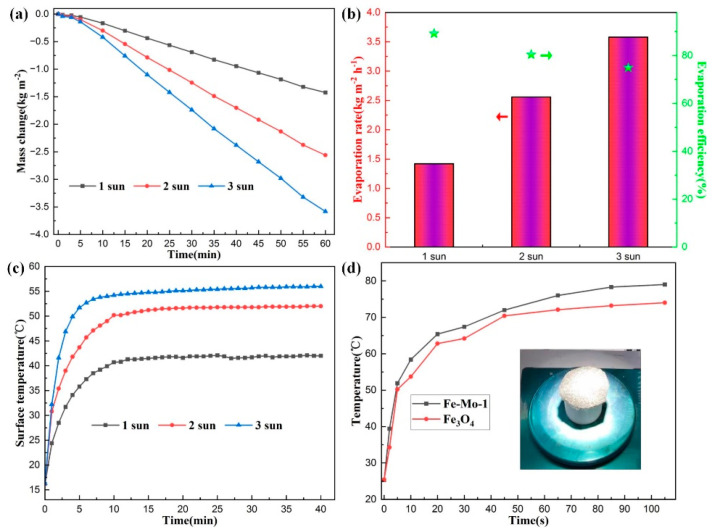
(**a**) The mass change of the Fe-Mo-1 sample with time under 1, 2, and 3 sun irradiation, respectively. (**b**) Evaporation rate and evaporation efficiency of Fe-Mo-1 under 1, 2, and 3 sun irradiation, respectively (The bar graph corresponds to the evaporation rate on the left axis and the pentagram corresponds to the evaporation efficiency on the right axis). (**c**) The surface temperature trend diagram of Fe-Mo-1 with time under 1, 2, and 3 sun irradiation. (**d**) Time dependent trend graph of surface temperature under 1 sun irradiation for Fe-Mo-1 (dried) and Fe_3_O_4_ (dried); inset is the physical picture of the sample at the time of experiment.

Figure 5a shows the curves of seawater mass changes in 60 min under 1, 2, and 3 sun irradiations using the Fe-Mo-1 sample. It can be seen from the figure that the greater the light intensity, the greater the loss of seawater quality. Under 1, 2, and 3 sun irradiation, the mass change for 60 min is 1.42 kg m^−2^, 2.56 kg m^−2^, and 3.58 kg m^−2^, respectively, indicating that the mass change is not linear with light intensity. According to the calculation, the seawater evaporation rates under 1, 2, and 3 sun irradiation are 1.42 kg m^−2^ h^−1^, 2.56 kg m^−2^ h^−1^, and 3.58 kg m^−2^ h^−1^, respectively, and the evaporation efficiency is 89.2%, 80.4%, and 74.9%, respectively (Figure 5b). With an increase in light intensity, the evaporation efficiency decreases. This is mainly because the stronger the light intensity, the higher the temperature of the sample surface will be, and then the intensity of thermal convection and thermal radiation will increase. At the same time, the more reflection of solar light there will be, the stronger the solar light intensity will be, and the lower the evaporation efficiency will be [35]. Figure 5c shows the trend of surface temperature variation with time during the evaporation of the Fe-Mo-1 sample under 1, 2, and 3 sun irradiation, respectively. The temperature of the sample Fe-Mo-1 surface has a similar overall change trend under different sun irradiations; the temperature rises very fast in the first 5 min, rises slowly between 5 min and 10 min, and tends to stabilize after 10 min. After the temperature of the sample surface stabilized, it was found that the temperature of the sample surface under 2 and 3 sun irradiations was higher than that of the sample surface under 1 sun irradiation by 10 °C and 14 °C, respectively; nor was the magnitude of the rise in visible temperature and light intensity linear, further confirming that the stronger the light intensity, the lower the photothermal conversion efficiency would be.

**Figure 6:**
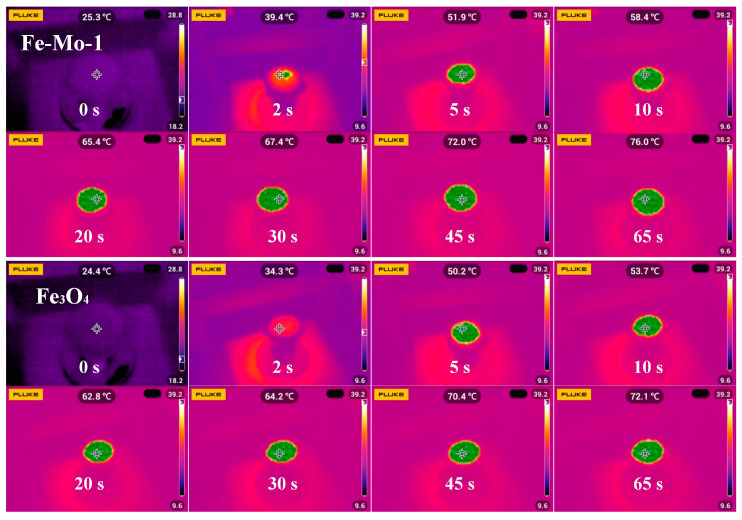
Infrared radiation images of Fe-Mo-1 (dried) and Fe_3_O_4_ (dried) at different times during the experiment.

To further verify that the photothermal conversion efficiency of the Fe-Mo-1 compound synthesized in this work is higher than that of Fe_3_O_4_, the two dried samples were placed under 1 sun irradiation for testing, respectively, and the real-time trend graphs of the changes in surface temperature over time for the two samples were taken as shown in Figure 5d, and the inset is the physical picture at the time of sample testing. The temperature on the surface of both samples in the first 30 s rises very quickly, followed by a temperature rise that starts to slow down, and the temperature starts to approach a plateau after 80 s. Overall, the sample surface temperature after stabilization of Fe-Mo-1 is higher than that of Fe_3_O_4_ by approximately 5 °C, confirming the better photothermal conversion efficiency of Fe-Mo-1. Figure 6 presents the infrared radiation images of the two samples at different times.

The main reasons for the good photothermal conversion rate of Fe-Mo-1 are as follows: (1) Both MoS_2_ and Fe_3_O_4_ have a strong photothermal conversion performance themselves, although the smooth MoS_2_ surface reflects a large amount of sunlight and reduces its photothermal conversion performance; Fe_3_O_4_ nanospheres attached to the MoS_2_ nanosheets increase the specific surface area of the composite sample greatly, also reducing the reflection of sunlight and reducing heat conduction and thermal radiation loss, increasing the photothermal conversion efficiency [36]. (2) The sample was evenly spread on cotton, which has strong water absorption and can transmit enough seawater to the sample in real time to complete evaporation [37].

### 2.3. Water Treatment Performance

**Figure 7:**
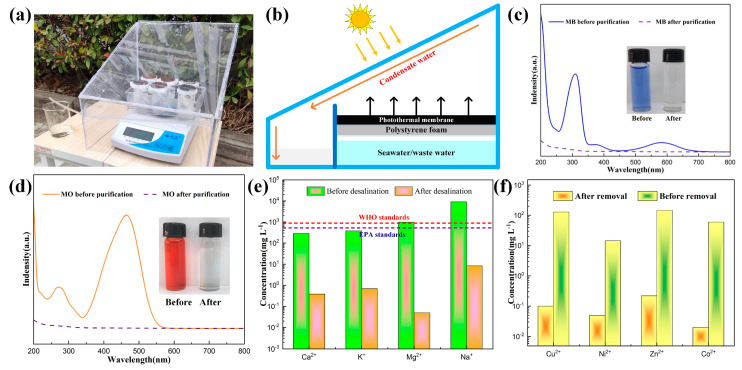
(**a**) Physical map of the SSG apparatus for outdoor measurements. (**b**) Schematic diagram of the outdoor SSG apparatus. (**c**) Solar absorption spectra before and after MB solution purification; inset shows the photos before and after MB solution purification. (**d**) Solar absorption spectra before and after MO solution purification; inset shows the photos before and after MO solution purification. (**e**) Comparison of ion concentrations in a simulated seawater solution before and after desalination. (**f**) Comparison of the concentrations of heavy metal ions in the sewage solution before and after purification.

Figure 7a shows the physical map for the outdoor SSG test setup in the laboratory, below which an electronic weight is placed, which is used to measure the mass change of water during the experiment. The schematic diagram of the test setup is shown in Figure 7b; the Fe-Mo-1 sample was placed on the polyethylene foam surface, using both ends of the cotton wick to connect the sample and seawater, respectively, which guaranteed that seawater could be adequately transmitted to the photothermal conversion layer. The role of polyethylene foam is to prevent direct sunlight and seawater/sewage contact, which can effectively reduce the loss of sunlight. Once the seawater evaporates, steam is pumped to the inside of the device, which is subsequently cooled into small drops that slip along the device into a beaker to complete the collection [38,39].

To verify the decontamination capacity of the SSG setup for sewage, the Fe-Mo-1 sample was used as the photothermal conversion layer. Figure 7c shows the sunlight absorption spectrum of the MB solution before and after purification, and the illustration shows the comparison of the MB images before and after purification. Figure 7d shows the absorption spectrum of sunlight before and after MO purification, and the illustration shows the comparison of MO before and after purification [40]. It can be found that the absorption rates of MO and MB are close to 0 after purification, and the purified sewage becomes transparent and clear; it is visible that the SSG apparatus has a good outcome for the purification of sewage. Figure 7e shows the comparison of the concentrations of Ca^2+^, K^+^, Mg^2+^, and Na^+^ in simulated seawater solution (a mass fraction of 3.5 wt%) before and after desalination. After desalination, the concentrations of several ions decreased by 3, 3, 4, and 3 orders of magnitude, respectively (Table 1). The ion concentration before desalination is far beyond the WHO standard, and after desalination, the ion concentration reaches the international standard for drinking water. The concentration of heavy metal ions Cu^2+^, Ni^2+^, Zn^2+^, and Co^2+^ in the sewage solution decreased by 3, 3, 3, and 4 orders of magnitude, respectively (Figure 7f, Table 2), indicating that the heavy metal ions in the sewage solution have been almost removed [41,42]. In addition, it has been shown that the sample and the SSG device fabricated in this work are an effective method for water purification.

**Figure 8:**
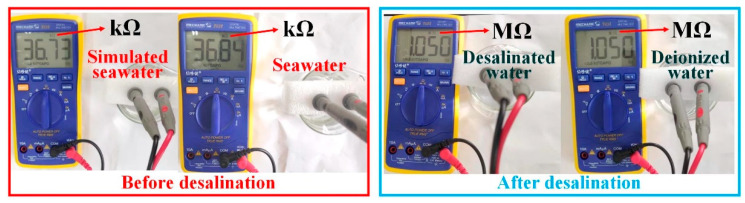
Comparison of resistance values of different water quality tested with a multimeter.

The quality of water can also be expressed using resistance values obtained from resistance tests performed at a constant distance with the multimeter electrode [43]. As shown in Figure 8, the resistances measured for the configured simulated seawater and the real seawater are 36.73 kΩ and 36.84 kΩ, respectively, indicating that the configured simulated seawater and the real seawater have similar ion concentrations. The simulated seawater and deionized water resistance after desalination were both measured to be 1.05 MΩ, which illustrated that seawater was effectively desalinated.

## 3. Experimental Section

### 3.1. Materials

Both ethylene glycol and diethylene glycol were purchased from Xilong Science Co., Ltd. (Shenzhen, China), and MoS_2_ was purchased from Shanghai Macklin Biochemical Co., Ltd. (Shanghai, China). Both sodium acetate and FeCl_3_·6H_2_O were purchased from Fuchen Chemical Reagent Co., Ltd. (Tianjin, China). Sodium citrate was purchased from Tianjin Guangfu Technology Development Co., Ltd. (Tianjin, China). Cotton was purchased from Anqing Nursing Co., Ltd. (Anqing, China). All materials are analytical reagents without further purification.

### 3.2. Preparation of Samples

#### 3.2.1. Preparation of Fe_3_O_4_

**Figure 9:**
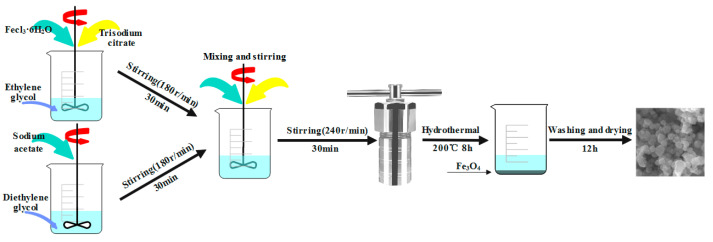
Production process of Fe_3_O_4_.

The Fe_3_O_4_ nanospheres were prepared by the hydrothermal method [44], and the preparation process is shown in Figure 9. First, FeCl_3_·6H_2_O (1.95 g, 7.2 mmol) and 0.1 g sodium citrate were added, respectively, to 40 mL of ethylene glycol solution and vigorously stirred with a magnetic stirrer (180 r/min) for approximately 30 min. Similarly, 4 g of sodium acetate was weighed and added to 40 mL of diethylene glycol, using a magnetic stirrer to stir vigorously (180 r/min) for about 30 min. Then the above two solutions were poured into the beaker and continued to be stirred with the magnetic stirrer (180 r/min) for about 30 min. After the fusion was uniform, the mixed solution was put in the reactor and reacted for 8 h at 200 °C. Finally, after the reactants were cooled to room temperature, the black precipitate was collected by the magnetic decantation method, rinsed 3 times with deionized water and anhydrous alcohol, and dried at 50 °C vacuum for 12 h, thus preparing nano Fe_3_O_4_.

#### 3.2.2. Preparation of Fe_3_O_4_@MoS_2_

The hydrothermal method used to prepare Fe_3_O_4_@MoS_2_ was similar to that used to prepare Fe_3_O_4_. FeCl_3_·6H_2_O (1.95 g, 7.2 mmol) and 0.1 g of sodium citrate were added to 40 mL of ethylene glycol, respectively, and vigorously stirred (180 r/min) for 30 min. Similarly, to contrast the evaporation performance of the materials synthesized by Fe_3_O_4_ and MoS_2_ at different mass ratios, the authors found the best evaporation performance at a mass ratio of 2:1 after several experiments. Therefore, 275 mg of MoS_2_ and 4 g of sodium acetate were weighed in 40 mL of diethylene glycol, vigorously stirred (180 r/min) for about 30 min and continued to be stirred (180 r/min) for 8 h after ultrasonic treatment for approximately 3 h, so that the mixed solution was evenly dispersed and uniform. The two solutions were mixed together, with continued stirring (240 r/min) for 30 min, and the mixture was transferred to a 100 mL Teflon lined stainless steel autoclave and reacted at 200 °C for 8 h using the hydrothermal method. After the reaction was complete, it was removed from the reactor and cooled to room temperature, and the black precipitate was collected using the magnetic precipitation method; it was washed repeatedly with deionized water and anhydrous ethanol three times and dried for 12 h in a vacuum environment of 50 °C; then Fe_3_O_4_@MoS_2_ was obtained, marked Fe-Mo-1. The Fe_3_O_4_ and MoS_2_ mass ratio of 5:1 was chosen as a comparison example, so the MoS_2_ mass in the reactant was changed to 110 mg and the rest was kept unchanged. The prepared sample was marked Fe-Mo-2.

### 3.3. Characterization of Samples

The scanning electron microscope was used to observe the microstructure of the sample (Hitachi Regulus 8100, Tokyo, Japan). X-ray diffraction (XRD) (Bruker D2 Phaser, Karlsruhe, Germany) was used for the components analysis of the sample. The scanning speed was 5°/min, and the scanning angle range was 10–80°. The solar absorption of the sample was measured by UV-Vis-NIR spectrophotometer (Shimadzu UV-3600, Tokyo, Japan).

### 3.4. SSG Experiment

**Figure 10:**
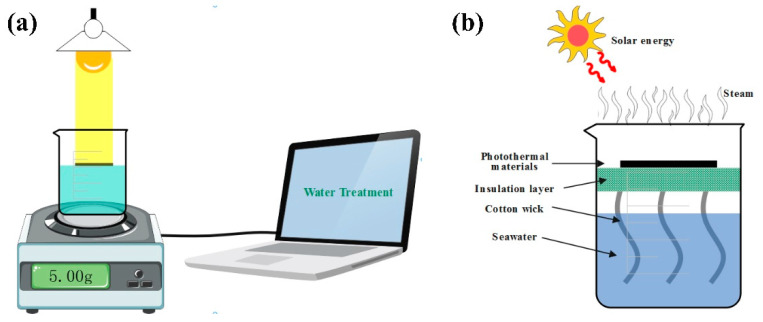
(**a**) Schematic diagram of SSG system experimental platform. (**b**) Schematic diagram of the photothermal conversion device.

The schematic diagram of the SSG experiment platform is shown in Figure 10a. The laptop was connected to a balance with automatic measurement data recording, and the quality information collected was transmitted to the laptop in real time. A beaker was placed above the balance and the liquid in the beaker was sewage/seawater to be vaporized. The schematic diagram of the photothermal conversion device is shown in Figure 10b. The photothermal conversion layer was the sample prepared in this work. The sample was placed on polyethylene foam which was suspended in the middle of the beaker as a heat insulation layer. The main function of the heat insulation layer was to block the contact between sunlight and seawater, to block heat radiation and heat convection, so that sunlight was absorbed by the material as much as possible and converted into heat energy for water evaporation, which makes the experimental results more accurate. The transfer of water between seawater and the photothermal conversion material used a cotton wick, which ensured that the liquid in the beaker could be continuously transported to the photothermal conversion layer for evaporation [45]. The xenon lamp (CEL-HXF300-T3, CEAULIGHT, Beijing, China) was used to simulate sunlight and an electronic scale (JS-A5, CEAULIGHT, Suzhou, China) was used to measure the evaporation quality of seawater in real time; the scale was connected to the computer and fed the measured data back to the computer for real-time recording. The infrared radiation imager (PTi120, Fluke, Shanghai, China) was used to measure the temperature of the sample surface and take infrared photos. The optical power meter (CEL-FZ-A, CEAULIGHT, Beijing, China) was used to calibrate the light intensity of xenon light.

### 3.5. SSG Test

By adjusting the light power of the xenon lamp, the sample was carried out under 1, 2, and 3 sun irradiation, and all experiments were completed at an ambient temperature of 20 °C and an ambient humidity of 60%. In the experiment, the liquid in the beaker was simulated seawater, and 150 mL of simulated seawater with a mass fraction of 3.5 wt% was prepared with sea salt (Yiery1, Yier, Guangzhou, China) and deionized water. The cotton piece was made into a round piece with a diameter of 4 cm, and a brush was used to evenly smear the sample on the cotton piece; it was placed on the polyethylene foam in the beaker, allowed to stand for half an hour while waiting for the seawater to wet the whole photothermal conversion layer through capillarity, and then the test was started. The electronic scale was used to measure the change in seawater quality in real time within one hour, and the infrared radiation imager was used to monitor the change in the surface temperature of samples under different light intensity and different times.

In the SSG experiment, the evaporation rate and the evaporation efficiency are the key parameters that reflect the performance of the prepared sample. The evaporation rate can be calculated by weighing the mass change of seawater during the experiment, and the evaporation efficiency can be further calculated. The evaporation rate of water (*v*) (kg m^−2^ h^−1^) is calculated as follows [46]:

(1) $$v=\frac{\Delta m}{\Delta t\times S}$$

In the above formula, Δ*m* refers to the difference between the mass change of seawater with and without light during the experiment. Δ*t* is the duration of the evaporation experiment. *S* refers to the surface area of the photothermal conversion layer, which is 4π cm^2^ in this experiment.

The evaporation efficiency (*η*) represents the photothermal conversion capacity of the sample. The higher the value, the higher the photothermal conversion efficiency of the sample. This is calculated using the following formula [47]:

(2) $$\eta =\frac{v\times h_{v}}{P_{0}}$$

In the above formula, *η* is the evaporation efficiency of seawater. *v* is the result calculated using Formula (1), which represents the evaporation rate of seawater. *h_v_* represents the total evaporation enthalpy of water; referring to the relevant literature, this value is 2260 kJ kg^−1^ [48]. *P*_0_ is the radiant light power of the sun, and the corresponding incident light power under 1~3 sun is 1~3 kW m^−2^, respectively.

### 3.6. SSG Water Treatment

The laboratory used ICP-OES/MS (Agilent 5110, Santa Clara, CA, USA) to measure the concentration of Cu^2+^, Ni^2+^, Zn^2+^, Co^2+^ ions before and after sewage purification and the concentration of Na^+^, Mg^2+^, K^+^, Ca^2+^ ions before and after simulated seawater desalination. The sewage used in the experiment was simulated sewage (10 mg L^−1^ MB and 10 mg L^−1^ MO) prepared with organic dyes, and the seawater was prepared with sea salt according to the standard of 3.5 wt% mass fraction. The UV-Vis-NIR spectrophotometer (UV-3600, Shimadzu, Tokyo, Japan) was used to detect the light absorption performance of organic dyes and then judge the purification effect of the SSG device on organic dyes. The digital multimeter (V96C, Mechanic, Shenzhen, China) was used to measure resistance before and after simulated seawater desalination, and then judge whether the seawater desalination experiment achieved the expected effect.

## 4. Conclusions

In this work, the composite material with Fe_3_O_4_ nanospheres attached to the surface of the MoS_2_ nanosheet was synthesized by the hydrothermal method. The composite material has a larger specific surface area, an average light absorption rate of 97.11%, and a higher light absorption ability. Under one sun irradiation, the evaporation rate and efficiency were 1.42 kg m^−2^ h^−1^ and 89.18%, respectively, which was better than most reported materials. After 10 cycles of testing, it was observed that the sample has high stability. In addition, in this work an outdoor SSG test device was also made. Through the test, it was found that purified seawater and sewage reached the international drinking water standard. In general, this work provides new ideas for sustainable and efficient freshwater production.

## Figures and Tables

**Table 1:** Concentrations of Ca^2+^, K^+^, Mg^2+^, and Na^+^ ions in solutions before and after desalination.

Ion Type	Ca^2+^	K^+^	Mg^2+^	Na^+^
Ion concentration before desalination (mg/L)	286	380	990	9000
Ion concentration after desalination (mg/L)	0.38	0.68	0.05	8.37

**Table 2:** Concentrations of Cu^2+^, Ni^+^, Zn^2+^, and Co^2+^ ions in solution before and after purification.

Ion Type	Cu^2+^	Ni^+^	Zn^2+^	Co^2+^
Ion concentration before purification (mg/L)	128	14.5	147	60
Ion concentration after purification (mg/L)	0.1	0.05	0.22	0.02
